# Incidence and risk factors of neurological complications during posterior vertebral column resection to correct severe post-tubercular kyphosis with late-onset neurological deficits: case series and review of the literature

**DOI:** 10.1186/s13018-018-0979-7

**Published:** 2018-10-26

**Authors:** Wenbin Hua, Xinghuo Wu, Yukun Zhang, Yong Gao, Shuai Li, Kun Wang, Xianzhe Liu, Shuhua Yang, Cao Yang

**Affiliations:** Department of Orthopaedics, Union Hospital, Tongji Medical College, Huazhong University of Science and Technology, Wuhan, 430022 China

**Keywords:** Post-tubercular kyphosis, Kyphosis, Late-onset neurological deficits, Posterior vertebral column resection, Neurological complication

## Abstract

**Background:**

Severe post-tubercular kyphosis with late-onset neurological deficits is difficult to treat, with high risk of neurological complications. This study retrospectively evaluates the efficacy and safety of posterior vertebral column resection (PVCR) for treating severe post-tubercular kyphosis with late-onset neurological deficits.

**Methods:**

From January 2012 to December 2015, 13 patients with severe post-tubercular kyphosis underwent PVCR. All these patients were of late-onset neurological deficits. The operative time, blood loss, preoperative and postoperative kyphotic angles, sagittal vertical axis (SVA), neurological status, and complications were recorded. The preoperative and postoperative Oswestry Disability Index (ODI) scores and visual analog scale (VAS) scores for back pain were compared. The American Spinal Injury Association (ASIA) grading system was used to evaluate neurological function.

**Results:**

The mean postoperative follow-up period was 28.6 months. The mean operative time was 388 ± 46 min. The mean blood loss was 2554 ± 1459 ml. The mean preoperative and postoperative kyphotic angles were 93.7 ± 14.4° and 31.7 ± 7.3°, respectively, with a mean correction of 62.0 ± 13.8°. The mean preoperative and postoperative SVA were 43.2 ± 44.4 mm and 17.8 ± 16.2 mm, respectively. The mean ODI score improved from 56.3 ± 5.1 preoperatively to 18.3 ± 18.5 at last follow-up. The mean VAS score improved from 6.4 ± 1.8 preoperatively to 1.8 ± 0.8 at last follow-up. Two cases had spinal cord injuries, including one complete paraplegia and one incomplete paraplegia, and a total neurological complication rate of 15.4%. The risk factors for neurological complications were summarized.

**Conclusions:**

Severe post-tubercular kyphosis with late-onset neurological deficits can be corrected by PVCR carefully and properly to prevent neurological complications. In many cases with stenosis adjacent to the angular kyphosis, sufficient decompression of the spinal cord at the segments with stenosis is necessary before correcting the kyphosis.

## Background

Tuberculosis (TB) of the spine is one of the most common causes of kyphosis [1–3]. Although anti-tuberculosis drugs are highly effective for controlling tubercular infection, 3–5% of patients with this condition have severe progression of the disease, with a kyphotic angle greater than 60°, leading to “buckling collapse” [2, 4]. Severe kyphosis results in back pain, spinal cord compression, cardiopulmonary dysfunction, costopelvic impingement, and cosmetic concerns [1].

The major complications of spinal tuberculosis are kyphosis, neurological deficits, or paraplegia. Neurological deficits can be caused by tuberculosis infection and the progression of post-tubercular kyphosis [1]. Early-onset neurological deficits or paraplegia is usually found in the active stages of tuberculosis, which should be treated with chemotherapy and surgery [5]. Late-onset neurological deficits or paraplegia usually develops due to the progression of kyphosis, which can be prevented with early stabilization surgery or a combined anterior-posterior procedure or posterior three-column osteotomy [5, 6]. Besides, late-onset neurological deficits may be related to a lesion cephalad or caudal from the kyphosis [7].

In cases with severe kyphosis, osteotomies are essential to reconstruct the sagittal alignment. Various techniques have been described for correcting post-tubercular kyphosis, including pedicle subtraction osteotomy [8], closing-opening wedge osteotomy [9, 10], and vertebral column resection [11–18]. Due to the complexity of severe post-tubercular kyphosis and accompanying late-onset neurological deficits, posterior vertebral column resection (PVCR) seems to be the most effective surgery that allows for sufficient correction of the deformity and decompression of the spinal cord [12–14, 16, 17].

Even though excellent correction of the kyphosis could be achieved with PVCR [12–14, 16, 17], it may be of high risk of neurological complications in cases of severe post-tubercular kyphosis with late-onset neurological deficits. To the best of our knowledge, there is a rare study about PVCR for correcting severe post-tubercular kyphosis with late-onset neurological deficits. Besides, severe post-tubercular kyphosis with late-onset neurological deficits caused by stenosis adjacent to the angular kyphosis is also rarely reported. In the present study, we summarized the clinical efficacy, incidence, and risk factors for neurological complications of PVCR to correct severe post-tubercular kyphosis with late-onset neurological deficits.

## Methods

### Patient population

From January 2012 to December 2015, 13 patients (9 male, 4 female; mean age 40.7 years, range 31–54 years) with severe post-tubercular kyphosis (kyphotic angle > 60°) underwent PVCR in our department. This study was conducted in accordance with the guidelines of the Declaration of Helsinki and was approved by the ethics committee of our hospital. Written informed consents were obtained from all the patients. Each patient included had a history of spinal tuberculosis in childhood or adolescence and was treated by chemotherapy. Each patient included had back pain and late-onset neurological deficits, with preoperative neurologic symptoms, including leg weakness (13 cases) and additional leg pain (4 cases). One case (No. 8) must walk with a cane. No case was found with sphincter dysfunction. Details of preoperative deformity and neurologic status of included patients were summarized in Tables 1 and 2. Patients with active tuberculosis, congenital kyphosis, kyphosis caused by other diseases, or early-onset neurological deficits were excluded.

**Table 1:** Details of the deformity and osteotomy

Patient no.	Age (years)	Gender	Time between first TB infection and surgery (years)	Affected segments	Number of affected segments	Deformity sites	Resected segments	Number of resected segments	Instrumented segments
1	47	Male	42	T9-L3	7	Thoracolumbar	T12-L1	2	T7–9, L3-L4
2	40	Female	23	T7-T9	3	Thoracic	T8	1	T5-T6, T10-T11
3	45	Male	31	T10-L1	4	Thoracolumbar	T11-T12	2	T8-T10, L2-L4
4	47	Male	29	T8-T10	3	Thoracic	T9	1	T7-T8, T10-T11
5	42	Male	32	T3-T7	5	Thoracic	T5	1	C6,T1-T3, T8–11
6	37	Male	34	T5-T10	6	Thoracic	T7-T8	2	T2-T5, T10-L1
7	50	Male	45	T10-L2	5	Thoracolumbar	T11-L1	3	T8-T10, L3, L4
8	54	Female	53	T8-L2	7	Thoracolumbar	T11-T12	2	T6-T9, L2-L4
9	32	Male	21	T8-T10	3	Thoracic	T9	1	T5-T7, T11-L1
10	53	Female	49	T11-L2	4	Thoracolumbar	T12	1	T9-T11, L2-L4
11	42	Male	27	T6-T9	4	Thoracic	T7-T8	2	T4-T6, T9-T11
12	35	Male	30	T9-T12	4	Thoracolumbar	T10-T11	2	T6-T8, L1-L3
13	31	Female	15	T10-T12	3	Thoracolumbar	T12	1	T9-T11, L1-L2
Mean	40.7 ± 11.0	–	–	–	4.5 ± 1.4	–	–	1.6 ± 0.6	–

*TB* tuberculosis

**Table 2:** Details of late-onset neurological deficits

Patient no.	Angular kyphosis	Intervertebral disc degeneration	Calcification of ligamentum flavum	ASIA grade		
				Pre-op	Immediate post-op	Last follow-up
1	+	+ (T8/9)	+ (T8/9)	D	A	A
2	+	−	−	D	D	E
3	+	−	−	D	D	D
4	+		+ (T7/8, T10/11)	D	D	E
5	+	−	−	D	D	E
6	+	−	−	D	C	D
7	+	+ (T10/11)	+ (T10/11)	D	D	E
8	+	−	+(T10/11)	C	C	D
9	+	−	−	D	D	E
10	+	−	−	D	D	E
11	+	−	−	D	D	D
12	+	+ (T9/10)	+ (T9/10)	D	D	D
13	+	−	−	D	D	E

*ASIA* American Spinal Injury Association, *pre-op* pre-operation, *post-op* post-operation

### Radiographic assessment

Full-length spine radiographs (i.e., those that included the whole spine and pelvis) of patients standing in a neutral, unsupported position were taken preoperatively, immediately postoperatively, and at the last follow-up. Computed tomography (CT) was used to assess deformity and stenosis adjacent to the angular kyphosis. Magnetic resonance imaging (MRI) was used to assess deformity and stenosis adjacent to the angular kyphosis, evaluate the status of the spinal cord, and exclude intraspinal lesions.

The kyphotic angle was defined as the angle between the superior endplate of the first normal vertebrae above the collapsed segments and the inferior endplate of the first normal vertebrae below the collapsed segments [12]. The postoperative kyphotic angle was measured at the same segments. Sagittal vertical axis (SVA) was defined as the distance between the C7 plumb line (C7PL) and the posterior-superior corner of S1, and defined as positive if the C7PL was anterior to the posterior-superior corner of S1, or negative if the C7PL was posterior to the posterior-superior corner of S1 [19, 20]. Thoracic kyphosis and lumbar lordosis were not used to evaluate the sagittal balance of the whole group because of the destruction of the thoracolumbar junction in most cases.

### Surgical technique

PVCR was performed in each case to correct the deformity and improve the neurologic status. The osteotomy should be performed at the apex of the deformity. Spinal cord function was continuously monitored with somatosensory-evoked potentials (SEP) and motor-evoked potentials (MEP). Wake-up tests should be performed in necessary.

After administering general anesthesia, the patients were placed in prone position. A midline incision was made to expose the vertebrae to be fixed as far as the transverse processes. Transpedicular screws were implanted at least two segments above and below the resected segments. After performing facetectomy and laminectomy at the osteotomy sites, the spinal cord was sufficiently exposed and decompressed. The costal heads were transected together with the costotransverse joints, with the pleura carefully protected [9]. Two temporary titanium rods were applied on the opposite sides to maintain spinal stability when performing osteotomy and the following correcting procedures. The deformity was corrected step-by-step with one of the rods fixed. Sagittal alignment was restored by moderately compressing the posterior column and distracting the anterior column. Thoracic nerve roots at the osteotomy segments were sacrificed by ligation. Meanwhile, the spinal cord and nerve roots were examined carefully to avoid excessive spinal cord tension, shrinkage, or compression near the osteotomy sites. The prevertebral vessels were protected by S-shaped retractor. The bleeding from the intravertebral venous plexus could be controlled by fluid gelatin. In cases with stenosis at the adjacent segments of the apex, the segments with stenosis should be decompressed before correcting the deformity. After performing the correcting procedures, the temporary rods were replaced by permanent rods. A titanium cage with autogenous iliac crest bone inside was applied to support the vertebral column and prevent excessive shortening of the spine. Posterolateral allograft (Aorui, China) was applied to achieve posterior column fusion of the spine. The wound was closed in layers over drains.

### Data collection

Serial radiographs were obtained, and clinical examination was performed at 3, 6, 12, and 24 months postoperatively. Each patient underwent at least 24 months follow-up. The preoperative and postoperative kyphotic angles and SVA were documented. The Oswestry Disability Index (ODI) scores and visual analog scale (VAS) scores for back pain were used to assess the back pain before surgery and at follow-up visits. Intraoperative and postoperative neurological complications and general complications were recorded. The American Spinal Injury Association (ASIA) grading system was used to evaluate neurological function.

### Statistical analysis

SPSS version 22.0 (SPSS Inc., Chicago, IL, USA) was used for statistical analysis. All data were presented as the mean ± standard deviation. The Wilcoxon signed-rank test was used to compare preoperative and postoperative data. A *P* value less than 0.05 was considered statistically significant.

## Results

### Clinical outcome

**Fig. 1:**
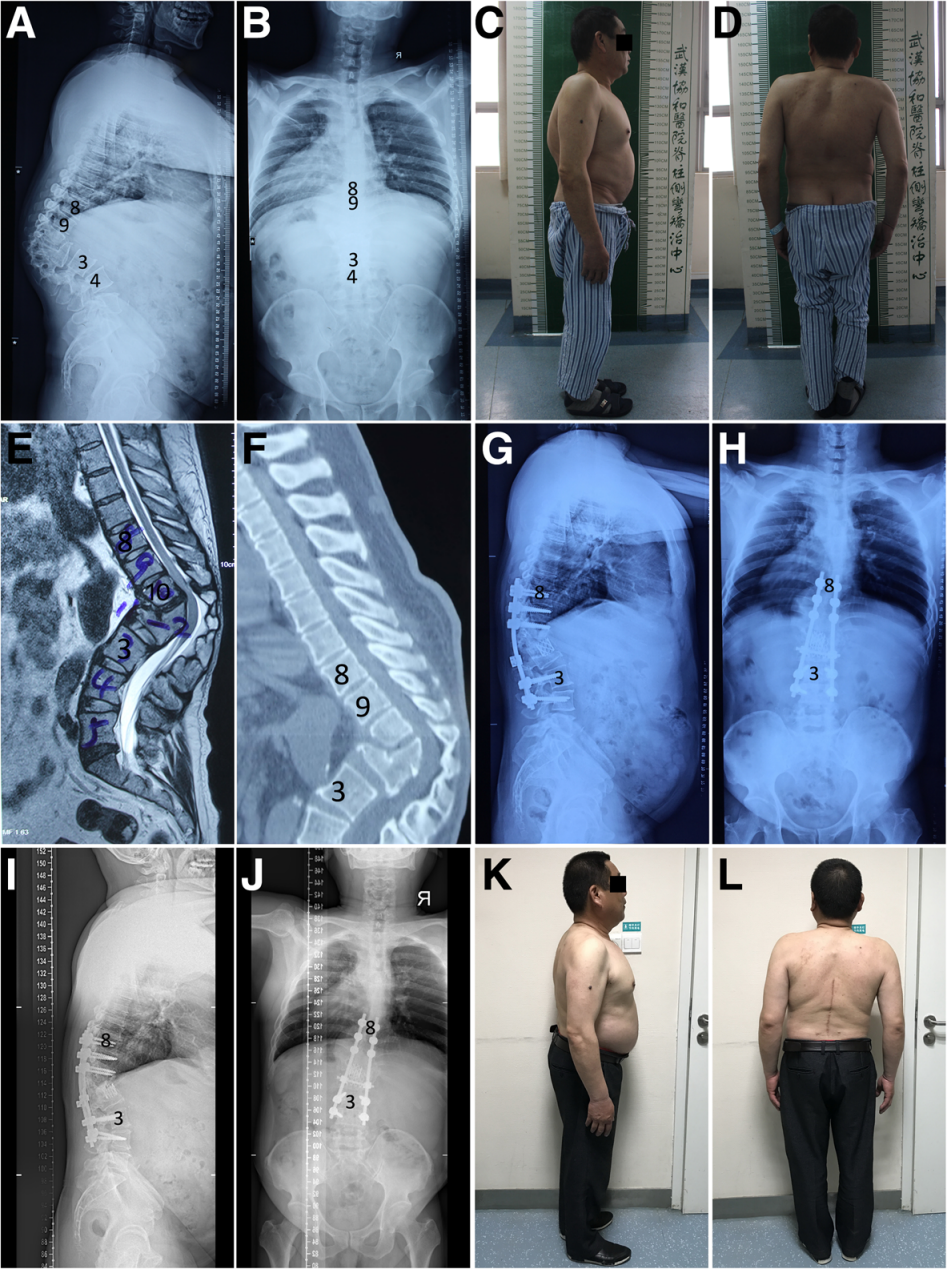
Standing radiographs and images of a 50-year-old male patient with severe post-tubercular kyphosis. **a**, **b** Preoperative lateral and antero-posterior radiographs, demonstrating severe angular kyphosis, with preoperative kyphotic angle 86° and sagittal vertical axis 45 mm. **c**, **d** Preoperative appearance in side and posterior view. **e** Preoperative magnetic resonance imaging scanning demonstrates stenosis adjacent to the angular kyphosis and severe spinal compression. **f** Preoperative computed tomography scanning demonstrates severe angular kyphosis and “buckling collapse.” **g**, **h** Postoperative lateral and antero-posterior radiographs, showing correction of thoracolumbar kyphosis after PVCR of T11, T12, and L1, with postoperative kyphotic angle 27° and sagittal vertical axis 33 mm. **i**, **j** Postoperative lateral and antero-posterior radiographs, showing excellent correction of thoracolumbar angular kyphosis and osteotomy site fusion at the 24-month follow-up, with kyphotic angle 29° and sagittal vertical axis 42 mm. **k**, **l** Postoperative appearance in side and posterior view

**Fig. 2:**
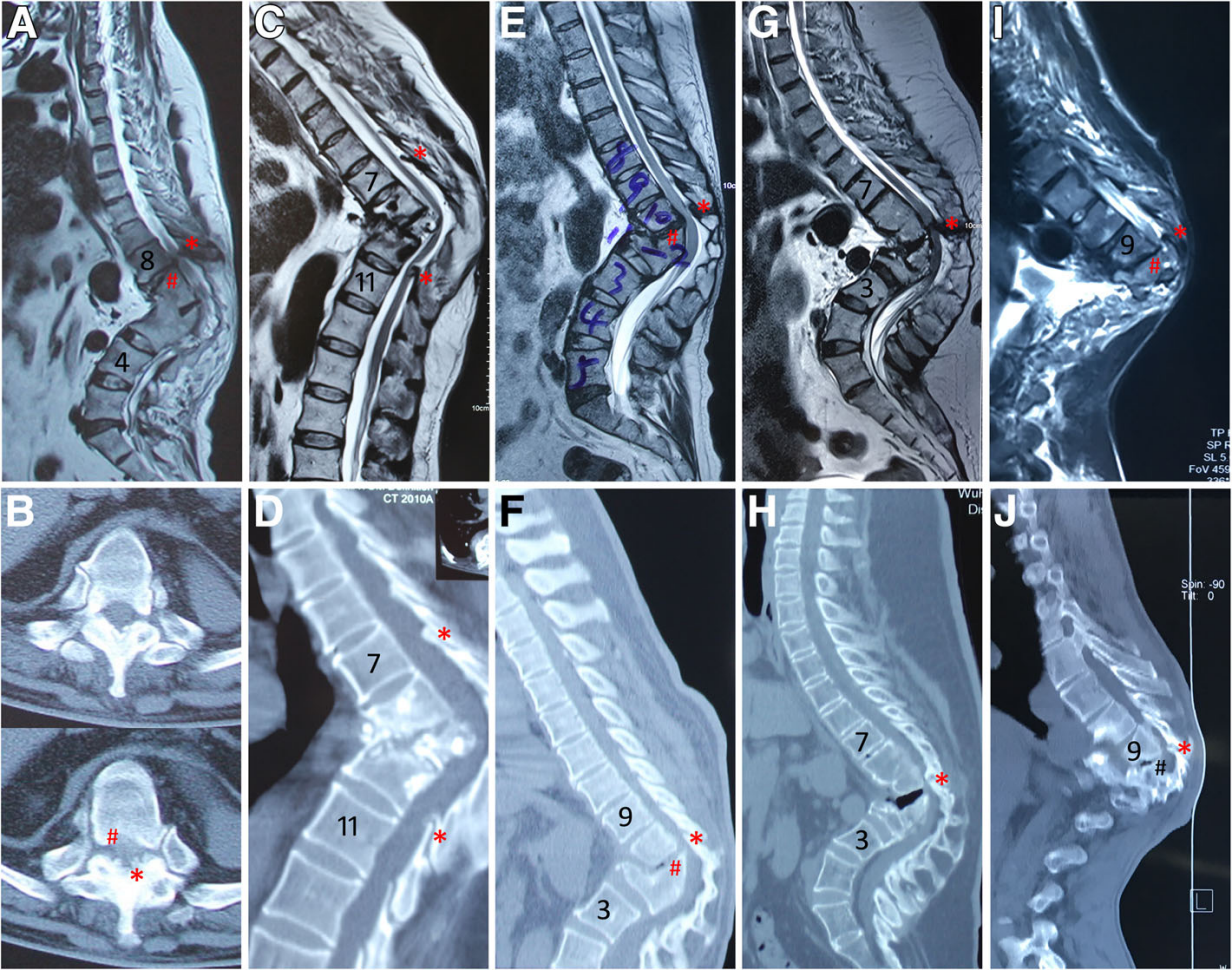
Causes of late-onset neurological deficits according to the lesions. **a**, **b** Preoperative magnetic resonance image (MRI) and computed tomography (CT) scanning demonstrate severe angular kyphosis, “buckling collapse” from T9 to L3, intervertebral disc herniation and calcified ligamentum flavum at T8/9, and severe spinal cord compression. **c**, **d** Preoperative MRI and CT scanning demonstrate severe angular kyphosis from T8 to T10, calcified ligamentum flavum at T7/8 and T10/11, and severe spinal cord compression. **e**, **f** Preoperative MRI and CT scanning demonstrate severe angular kyphosis, “buckling collapse” from T10 to L2, intervertebral disc herniation and calcified ligamentum flavum at T10/11, and severe spinal cord compression. **g**, **h** Preoperative MRI and CT scanning demonstrate severe angular kyphosis, “buckling collapse” from T8 to L2, calcified ligamentum flavum at T10/11, and severe spinal cord compression. **i**, **j** Preoperative MRI and CT scanning demonstrate severe angular kyphosis, intervertebral disc herniation and calcified ligamentum flavum at T9/10, and severe spinal cord compression. *Calcified ligamentum flavum at the segments adjacent to the angular kyphosis; ^#^disc herniation at the segments adjacent to the angular kyphosis

The baseline demographic details of the deformity and osteotomy of these patients are summarized in Table 1 (Fig. 1). Details of late-onset neurological deficits, including preoperative status and postoperative improvement, are summarized in Table 2 (Fig. 2).

The mean postoperative follow-up period was 28.6 months (range, 24–48 months). The mean operative time was 388 ± 46 min (range, 300–450 min). The mean blood loss was 2554 ± 1459 ml (range, 800–6800 ml). The mean kyphotic angle improvement is summarized in Table 3. The mean SVA improvement is summarized in Table 3. Details of the improvement of clinical measurements, including ODI score and VAS score, are summarized in Table 4.

**Table 3:** Details of the correction of kyphotic angle and sagittal vertical axis

Patient no.	Kyphotic angle/°					SVA/mm				
	Pre-op	Immediate post-op	Correction	Last follow-up	Loss of correction	Pre-op	Immediate post-op	Correction	Last follow-up	Loss of correction
1	90	33	57	35	2	45	21	24	25	4
2	75	25	50	27	2	31	10	21	12	2
3	102	38	64	41	3	69	15	44	16	1
4	80	39	41	42	3	14	42	−28	36	−6
5	85	22	63	23	1	42	39	3	51	12
6	106	45	61	55	10	−5	31	−36	62	31
7	86	27	59	29	2	45	33	12	42	9
8	98	24	74	30	6	118	5	113	−28	−33
9	86	36	50	40	4	44	12	30	17	5
10	94	28	66	33	5	60	11	49	19	8
11	107	40	67	43	3	43	14	29	20	6
12	128	30	98	36	6	109	18	91	22	4
13	81	25	56	28	3	−54	−19	35	8	27
Mean	93.7 ± 14.4	31.7 ± 7.3*	62.0 ± 13.8	35.5 ± 8.6*	3.8 ± 2.4	43.2 ± 44.4	17.8 ± 16.2^#^	29.8 ± 40.9	23.2 ± 22.2^#^	5.4 ± 15.3

*SVA* sagittal vertical axis, *pre-op* pre-operation, *post-op* post-operation. *P = 0.001 compared with pre-op; ^#^*P >* 0.05 compared with pre-op

**Table 4:** Details of the improvement of clinical measurements

Patient no.	ODI score		VAS score	
	Pre-op	Last follow-up	Pre-op	Last follow-up
1	52	78	7	3
2	54	8	4	1
3	54	24	6	3
4	46	10	4	1
5	60	12	8	2
6	56	20	7	2
7	52	10	4	1
8	66	18	9	3
9	60	12	8	1
10	54	12	6	2
11	60	14	9	2
12	58	10	5	2
13	60	10	6	1
Mean	56.3 ± 5.1	18.3 ± 18.5*	6.4 ± 1.8	1.8 ± 0.8^#^

*ODI* Oswestry Disability Index, *VAS* visual analog scale, *pre-op* pre-operation, *post-op* post-operation. *P = 0.002 compared with pre-op; ^#^*P =* 0.001 compared with pre-op

### Neurological complications

Five cases had SEP or MEP changes during surgery. Two of them were confirmed to be spinal cord injuries (15.4%), of which one developed permanent complete paraplegia (ASIA grade A), the other developed incomplete paraplegia (ASIA grade C) and recovered (ASIA grade D) at the 6-month follow-up after surgery. Another three cases had temporary SEP or MEP changes during surgery without spinal cord injuries. The neurological function of the remaining cases improved during follow-up (Table 2).

For the two cases of spinal cord injuries, the possible causes were investigated. In the first case, SEP and MEP disappeared during surgery, with permanent complete spinal cord injury after surgery. Accompanying stenosis at the segments adjacent to the angular kyphosis was confirmed by preoperative MRI scanning, but the stenosis was not decompressed before correcting the kyphosis. Then, the spinal cord was compressed further at the segment with stenosis during the correcting procedures. In the later four cases with stenosis adjacent to the angular kyphosis, similar spinal cord injuries were avoided by sufficient decompression of the stenosis before correcting the deformity. Another case of stationary SEP and no MEP was found with weakened muscle strength of one leg after surgery. This may be resulted from over-correction of the kyphosis. As a result, such cases should be moderately corrected to prevent neurological complications.

## Discussion

Due to the multiple fused vertebral bodies and severe spinal cord compression in cases of severe post-tubercular kyphosis, the destroyed anterior column should be completely resected [13]; therefore, PVCR should be performed to achieve adequate correction. By using PVCR, the mean kyphotic angle of post-tubercular kyphosis can be improved significantly, with a mean correction ranged between 40.5 and 80° (57.3–82.3%) in the sagittal plane [12–14, 16, 17]. In the present study, a mean correction of 62.0° (66.2%) was achieved. Because of preexisting severe spinal cord compression or distraction before surgery, all these patients included were of late-onset neurological deficits; complete correction of post-tubercular kyphosis is unnecessary and associated with a high risk of neurological complications. Therefore, nearly 50% improvement of the kyphotic angle may be relatively safe during the correction of post-tubercular kyphosis [12].

Even though PVCR is effective in restoring alignment of the whole spine in patients with severe kyphosis, PVCR has a high risk of neurological complications because of the aggressiveness of the procedure and its high technical demands [12–14, 16, 17, 21–24]. According to the literature, Cobb angle of the main curve, sharp and angulated deformity, dural buckling, compression of the spinal cord, preexisting neurologic dysfunction, spinal cord ischemia during the surgery, levels of osteotomy, and subluxation of the spinal column contribute to the high risk of neurological complications when correcting severe spinal deformities by PVCR [21–24].

Performing PVCR in patients with severe post-tubercular kyphosis seems to be more challenging because of the additional risk factors involved; indeed, Tuli [25] reported that worse prognosis and higher incidence of postoperative paraplegia were observed in tuberculosis patients with kyphotic angle larger than 60°. Multiple fused vertebral bodies, post-infectious fusion masses, and tethered dural sacs were common in most cases of severe post-tubercular kyphosis [6]. Healed bony bars, calcified caseous material, tissue fibrosis, and increased kyphosis may aggravate the compression and distraction, contributing to ischemia and atrophy of the spinal cord [7]. Due to long-term compression, the spinal cord is already at the limit of its tolerance; therefore, patients with late-onset neurological deficits may suffer further spinal cord injuries and have relatively poorer prognosis than those without neurological deficits during osteotomy [7].

According to previous studies, the rate of neurological complications after PVCR, including paraplegia and incomplete spinal cord injury, ranged between 0 and 17.1% [22, 24, 26–32]. However, the incidence of neurological complications for severe post-tubercular kyphosis after PVCR is 0–11.1% [12–14, 16, 17]. In the present study, the neurological complication rate after PVCR is higher than that in the literature. As a result, much more attention should be paid to the risk factors of neurological complications during the correction of severe post-tubercular kyphosis with late-onset neurological deficits.

In typical cases of severe post-tubercular kyphosis, two or more vertebrae are destroyed, leading to shortening or “buckling collapse” of the anterior column, without collapse of the posterior column height [4, 10]. Because of the accompanying severe spinal cord compression and distraction, preexisting neurologic dysfunction, and the high risk of potential neurological deficits, sufficient decompression of the spinal cord at the segments of angular kyphosis and the adjacent segments with stenosis may be necessary. The main goal in treating severe post-tubercular kyphosis should be to improve neurologic deficits, prevent further neurological lesions, moderately correct severe kyphosis, and restore alignment and stability of the whole spine.

Stretching or kinking of the spinal cord could occur during opening or closing wedge osteotomy [3, 9]. Besides, subluxation of the spinal column may occur during the correction procedures [16]. Therefore, two temporary titanium rods are essential to maintain the stability of the spine and prevent sudden subluxation of the spine after resection of the apical vertebrae. Moreover, a titanium cage is necessary to support the anterior column, maintain the stability of the spine, and prevent over-shortening of the spine and spinal cord. A balance between the amount of anterior column height restoration and posterior column shortening is essential to avoid spinal cord damage.

Stenosis adjacent to the angular kyphosis, which may be an important reason of late-onset neurological deficits and risk factor for neurological complications during PVCR, can be caused by intervertebral disc degeneration and/or calcification of ligamentum flavum at the adjacent segments. Mechanical stress may be the cause for intervertebral disc degeneration and calcification of ligamantum flavum at the adjacent segments [33]. Luk and Krishna [34] reported two cases with late-onset neurological deficits caused by spinal stenosis above healed tubercular kyphosis. Chen et al. [35] found ossification of the ligamentum flavum at segments adjacent to the kyphotic apex in six patients with thoracic tuberculosis. Ha et al. [7] reported ten cases with late-onset neurological deficits, including ossified ligament flavum (four), spinal stenosis (four), and intervertebral disc herniation (two). In the present study, five cases with late-onset neurological deficits were confirmed with stenosis at the segments adjacent to the apex of angular kyphosis (Table 2, Fig. 2).

Additional risk factors for neurological complications include affected segments, number of resected segments, and intraoperative hypotension, among others. Zeng et al. [13] reported that preoperative neurological function was worse in the thoracic group, and this may be related to higher sensitivity of spinal cord to either direct compression or tension over the kyphotic deformity in the thoracic segments. In the present study, two cases with multilevel PVCR were found with spinal cord injuries. Intraoperative hypotension must be prevented to minimize the risk of spinal cord ischemia. A mean blood pressure higher than 80 mmHg should be recommended to ensure the adequate perfusion of the spinal cord [16, 36].

Spinal cord monitoring is essential to ensure safety of the correction osteotomy during PVCR [16, 36, 37]. Therefore, any preoperative SEP and MEP changes during surgery should be paid attention to.

However, this study is limited by its relatively small sample size; more studies with a large patient group are necessary to evaluate the neurological complications after treatment of severe post-tubercular kyphosis with late-onset neurological deficits by PVCR.

## Conclusions

Even though PVCR is effective to restore alignment of the whole spine, our findings suggest that it is nevertheless associated with a high risk for neurological complications following its use in the management of severe post-tubercular kyphosis with late-onset neurological deficits.

## Abbreviations

ASIA: American Spinal Injury Association
C7PL: C7 plumb line
CT: Computed tomography
MEP: Motor-evoked potentials
MRI: Magnetic resonance imaging
ODI: Oswestry Disability Index
PVCR: Posterior vertebral column resection
SEP: Somatosensory-evoked potentials
SVA: Sagittal vertical axis
TB: Tuberculosis
VAS: Visual analog scale
